# Consequences of high-sensitivity troponin T testing applied in a primary care population with chest pain compared with a commercially available point-of-care troponin T analysis: an observational prospective study

**DOI:** 10.1186/s13104-015-1174-0

**Published:** 2015-06-03

**Authors:** Per O Andersson, Jan-Erik Karlsson, Eva Landberg, Karin Festin, Staffan Nilsson

**Affiliations:** Primary Health Care and Department of Medical and Health Sciences, Linköping University, Linköping, Sweden; Department of Internal Medicine, County Council of Jönköping, Jönköping, Sweden; Department of Medical and Health Sciences, Linköping University, SE-581 83 Linköping, Sweden; Department of Clinical Chemistry and Department of Clinical and Experimental Medicine, Linköping University, Linköping, Sweden; Primary Health Care and Department of Medical and Health Sciences, Linköping University, Norrköping, Sweden

**Keywords:** Primary health care, Acute coronary syndrome, Chest pain, Troponin T, Point-of-care testing

## Abstract

**Background:**

There is a demand for a highly sensitive and specific point-of care test to detect acute myocardial infarction (AMI). It is unclear if a high-sensitivity troponin assay will have enough discriminative power to become a decision support in primary care. The aim of this study was to evaluate a high-sensitivity troponin T assay performed in three primary health care centres in southeast Sweden and to compare the outcome with a point-of-care troponin T test.

**Methods:**

This study included 115 patients who consulted their general practitioner for chest pain, dyspnoea on exertion, unexplained weakness and/or fatigue in the last 7 days. Troponin T was analysed by a point-of-care test and a high-sensitivity method together with N-terminal pro-B-type natriuretic peptide (NT-proBNP) and creatinine. All patients were checked for AMI or unstable angina (UA) within 30 days of study enrolment. Univariate and multivariate logistic regression was carried out to examine possible connections between troponin T ≥ 15 ng/L, clinical variables and laboratory findings at baseline. In addition, 21 patients with troponin T ≥ 15 ng/L and no signs of AMI or UA were followed up for 2–3 years.

**Results:**

Three patients were diagnosed with AMI and three with UA. At the ≥ 15 ng/L cut-off, the troponin T method had 100% sensitivity, 75% specificity for AMI and a positive predictive value of 10%. The troponin T point-of-care test missed one case of AMI and the detection limit was 50 ng/L. Troponin T ≥ 15 ng/L was correlated to age ≥65 years (odds ratio (OR), 10.9 95% CI 2.28–51.8) and NT-proBNP in accordance with heart failure (OR 8.62 95% CI 1.61–46.1). Fourteen of the 21 patients, without signs of AMI or UA at baseline, still had increased troponin T at follow-up after 2–3 years.

**Conclusions:**

A high-sensitivity troponin T assay could become useful in primary care as a point-of-care test for patients <65 years. For patients older than 65–70 years, a higher decision limit than ≥15 ng/L should be considered and used in conjunction with clinical parameters and possibly with NT-proBNP.

## Background

Chest pain is a challenge for the general practitioner (GP) and is a symptom found in 1–3% of primary care patients [1, 2]. Chest pain can be caused by a wide range of illnesses and most are not of cardiac origin. Chest pain is due to ischaemic heart disease in 10–18% of cases, acute myocardial infarction (AMI) in 2–4% of cases, or unstable angina (UA) [3, 4], all of which require immediate attention. Cardiac troponin T (cTnT) is a biomarker specific for cardiac injury and an increased plasma concentration together with a dynamic course is one of the most important criteria for AMI used in hospital care [5]. Some authors have suggested that measurement of cTnT could also help to assess patients with chest pain in primary care [6–10]. In a previous study, we found that the use of a commercially available point-of-care test (POCT) for cTnT may reduce emergency referrals but at the cost of a number of missed cases of AMI or UA [11]. We found that the sensitivity of the POCT-cTnT test to diagnose an AMI was 67% and for AMI and UA together, only 29%, using the current detection level of 30 ng/L. This emphasizes the need for a more sensitive POCT troponin assay in primary care. However, there are no POCTs for high-sensitivity troponin T (hs-cTnT) available. The use of a potential hs-cTnT or cTnI test in primary care has some pitfalls. If the blood sample is drawn too early in the course of an AMI, the troponin value may still be below the decision limit and an AMI or UA may be overlooked. There is also a demand for a method with high specificity to avoid results above the decision level with no AMI. This is particularly important in primary care, because AMI is more often not the cause of chest pain in this population [3, 4]. Poor specificity of hs-cTnT has been an issue in several studies and falsely increased cTnT is common in older patients with decreased renal function [5, 12].

The aim of this study was to evaluate an hs-cTnT assay applied to a primary care population with chest pain and to compare the outcome with POCT-cTnT. Another aim was to investigate the persistence of hs-cTnT above the cut-off limit of 15 ng/L after 2–3 years in patients with no evidence of acute coronary syndrome.

## Methods

### Study design

An observational prospective study with follow-up was performed between May 2009 and January 2011 in 3 primary health care (PHC) centres in the county of Östergötland, situated in southeast Sweden. In the study published in 2013 by Nilsson et al. [11], samples from 128 patients with chest pain were investigated by POCT-cTnT. However, present study includes samples from 115 of these patients because in 13 cases the blood samples were lost (four due to a broken freezer, nine due to other administrative failures). One of these lost cases was diagnosed as UA.

### Data collection

In all three PHC centres, according to normal routines, all patients were given an appointment with their GP after calling the PHC centre and talking to a nurse; the nurse included all eligible patients according to the following inclusion criteria: chest pain, dyspnoea on exertion, unexplained weakness and/or fatigue commencing or becoming aggravated during the last 7 days; age ≥35 years. Exclusion criteria were as follows: patients with symptoms severely affecting their general condition; probable causes of chest pain other than cardiac, according to a nurse’s telephone assessment, e.g. costal fracture or gastroesophageal reflux. In addition to the patients included by the nurses, the GPs were asked to include eligible patients in conjunction with consultations.

Management of the patients was noted by the GPs on the case report form developed for the study. The following variables were used: current smoker; ongoing smoking or stopped less than 1 month ago; diabetes, on a diet or medication; hypertension, on medication currently or previously; hypercholesterolaemia, taking cholesterol-lowering medication; angina pectoris, effort-related chest pain; previous AMI, previous diagnosis of myocardial infarction, also silent myocardial infarctions with obvious changes found on an electrocardiogram (ECG) or echocardiography; coronary revascularization, previous percutaneous coronary intervention or coronary artery bypass graft operation; stroke and heart failure, previously diagnosed; aortic valve disease, insufficiency or stenosis of more than slight significance. A clinical diagnosis of renal failure was based on a plasma creatinine level of >200 µmol/L in men and >160 µmol/L in women. An ECG was registered and interpreted by the GPs in conjunction with their first assessment of each patient in the study.

The end point evaluation method is described elsewhere [11]. The cases of AMI and UA in the study were diagnosed in conjunction with the first visit. They could also be diagnosed within 30 days provided they were assessed as missed cases in primary care [11]. The diagnoses of AMI and UA were based on the current definitions [5].

### Follow-up of patients with hs-cTnT levels above 15 ng/L but without signs of AMI or UA

Twenty-six patients had hs-cTnT levels above 15 ng/L but no evidence of AMI or UA within 30 days after drawing the blood sample. Three of the patients had died by the follow-up. The remaining 23 were invited for a follow-up examination. One patient declined due to advanced cancer and one had moved to another county in Sweden. The remaining 21 accepted the invitation. The patients were asked to avoid physical exercise the day before the examination because earlier studies have shown that physical exercise or hard physical training can cause increased hs-cTnT levels [13]. Follow-up was performed at the PHC centre where the patient had chosen to be listed. The time after inclusion in the study varied between 25 and 42 months (average 34 months). In all 21 cases, the follow-up was performed by the same researcher.

All patients were asked the same questions from a special form that was produced exclusively for this study and the same procedure was used for everyone. Data on ECG, weight, height, body mass index, waist circumferences and blood pressure were collected. An ECG was registered and compared with the ECG that was performed at the start of the study. We searched for new findings, i.e. atrial fibrillation, ST elevation and pathologic q-waves. Blood pressure was taken from the right arm with the patient in a sitting position; patients rested for 5 min before the measurements were taken. Waist circumference was measured with the patient in a standing position during exhalation. Blood samples were collected by venipuncture and sent for analysis of cholesterol, creatinine, N-terminal pro-B-type natriuretic peptide (NT-proBNP) and hs-cTnT on the same day. The diagnosis of heart failure was based on impaired left ventricular function according to echocardiogram if performed earlier, and/or by clinical findings. Ten of the patients had undergone echocardiogram from 8 years to 6 months before the follow-up. At the consultation, the patients were asked about new symptoms since inclusion in the study, i.e. chest pain, dyspnoea, angina or exercise-related chest pain.

### Analytical methods

#### Blood samples

Blood was collected by venipuncture in vacuum tubes containing separating gel and lithium heparin (4 mL; Greiner Bio-One, Frickenhausen, Germany) or separating gel and silica coagulation activator (4 mL; Greiner Bio-One). After resting for 30–60 min, the tubes were centrifuged at 2,400×*g* for 5 min. Aliquots of serum and plasma were stored initially at −20°C for less than 1 month, after which the samples were transferred to −70°C and kept frozen until analysis.

#### POCT-cTnT

Troponin T was measured on the POCT instrument Cobas h232 (Roche Diagnostics, Mannheim, Germany). The detection limit was 0.03 µg/L (30 ng/L) and all values >0.03 µg/L (>30 ng/L) were regarded as positive according to the manufacturer’s recommendations. Further details about the method for POCT-cTnT are described elsewhere [11].

#### hs-cTnT

Cardiac troponin T was measured in plasma by immunochemical methods using detection based on luminescence on a Cobas e602 instrument (Roche Diagnostics, Mannheim, Germany). The method used for cTnT was a highly sensitive method with a limit of detection of 1 ng/L. A decision limit of ≥15 ng/L was used. This limit was based on the 99th percentile calculated for a healthy population, according to Roche [14]. The lot number of the reagent was 167,650 and of the calibrator 165,095. These are new revised lots. However, when patients were admitted to the hospital, the diagnosis was based on hs-cTnT values measured by the old non-revised lots, which, according to Roche, have recently been found to produce low results in the interval 3–20 ng/L [15]. The coefficient of variation (CV) for a 2-month period was 2.9% (level 24 ng/L) and 3.6% (level 1,600 ng/L) for hs-cTnT.

#### Other laboratory analyses

Creatinine was measured in plasma samples from all patients using a standardized method (Advia 1800; Siemens Healthcare Diagnostics, Deerfield, IL, USA). The method was based on Jaffe reagent and contained a rate-blanking measurement to compensate for interference from bilirubin and a correction for intercept due to pseudocreatinines. An estimated glomerular filtration rate (eGFR) was calculated based on the creatinine level using the modification of diet in renal disease equation [16].

N-terminal pro B-type natriuretic peptide (NT-proBNP) was measured in plasma samples from all patients at baseline and at follow-up. Analysis was performed on a Cobas e602 instrument (Roche Diagnostics). This assay is based on immunochemistry with 2 monoclonal antibodies and electrochemiluminescence detection.

Cholesterol was measured in plasma samples from patients at the follow-up. Analysis was performed on an Advia 1800 instrument (Siemens Healthcare Diagnostics) by a method with reagent and calibrator from the same company. The reference ranges used were 3.3–6.9 mmol/L (31–50 years) and 3.9–7.8 mmol/L (>50 years) [17].

### Statistical analysis

At baseline and at the follow-up, possible differences regarding demographics (age, gender), risk factors (smoking habits, diabetes, hypertension and hypercholesterolaemia), cardiovascular disease (angina pectoris, previous AMI, coronary revascularization, stroke, heart failure and aortic valve disease), ECG findings (sinus rhythm, atrial fibrillation), and laboratory findings (renal failure, eGFR, NT-proBNP, and potential causes of increased hs-cTnT) between patients with hs-cTnT levels above or below 15 ng/L were tested with the Pearson χ^2^ test and the Fisher exact test if the data were discrete. Continuous data were analysed with the Student *t* test if approximately normally distributed, otherwise the non-parametric Mann–Whitney test was used. Univariate and multivariate logistic regression was carried out to examine possible differences between high and low hs-cTnT levels at baseline regarding all the variables, except renal failure and potential causes of increased hs-cTnT (due to small numbers). In the logistic regression models, the continuous variable NT-proBNP was transformed into a dichotomous variable according to age and a high likelihood of heart failure, i.e. >450 ng/L (<50 years), >900 ng/L (50–75 years) and >1,800 ng/L (>75 years) [18].

**Figure 1:**
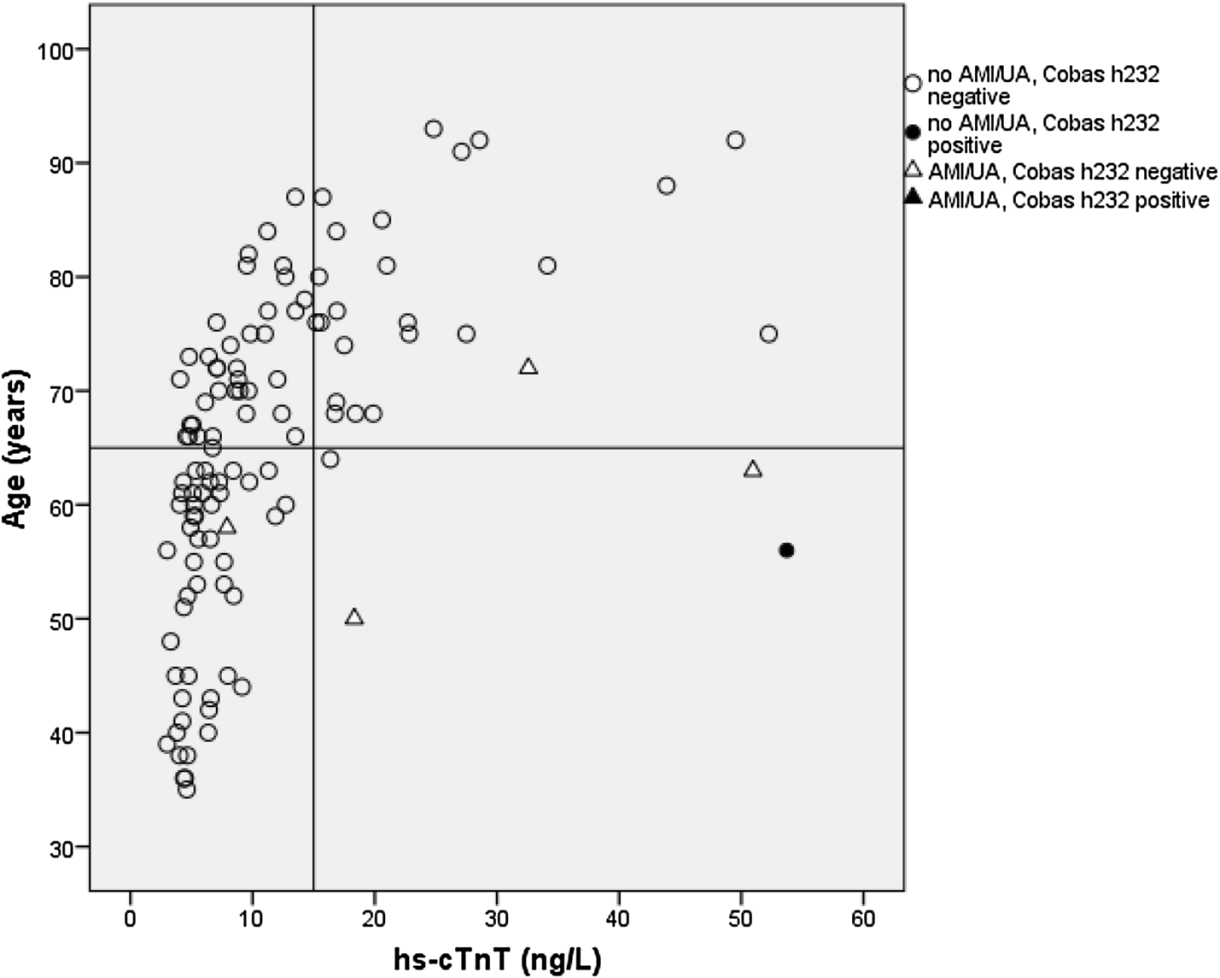
hs-cTnT values for 112 patients with chest pain in relation to age. The results from Cobas h232 were noted as positive in 4 cases (i.e. >30 ng/L). Three of these results are not shown in the figure because their hs-cTnT values were 204, 220 and 520 ng/L. Two of these patients had AMI.

Age was also transformed into a dichotomous variable; >65 years according to the results for emergency room patients reported by Hammarsten et al. [12] and our own findings (Figure 1). Data on angina pectoris, AMI and percutaneous coronary intervention/coronary artery bypass graft were combined into one variable due to small numbers. All variables were adjusted for sex and age in the univariate models, and those who had a *P* value <0.10 were considered for the multivariate model. A *P* value <0.05 was considered statistically significant.

### Ethics

The Regional Ethical Review Board in Linköping, Sweden (Dnr M101-09, T98-09 and Dnr 2010/211-32) approved the study. Written informed consent was obtained from all patients before enrolment in the study.

## Results

One hundred and fifteen patients were enrolled in the study. Only three had AMI and three had UA. Thirty-one patients had hs-cTnT >15 ng/L. The group with hs-cTnT >15 ng/L was on average 15 years older, more often male and more often diagnosed with heart failure or atrial fibrillation. They also had lower eGFR and higher NT-proBNP compared with those with hs-cTnT <15 ng/L (Table 1). hs-cTnT analysis had a much higher sensitivity for finding AMI (100 vs 67%) and the combination of AMI and UA (83 vs 33%) compared with POCT-cTnT. However, this increase in sensitivity was at the expense of greatly reduced specificity, from 98% to 75–76%. The positive predictive value (PPV) was only 10% for AMI and 16% for the combination of AMI and UA (Table 2). Besides a diagnosis of AMI or UA (odds ratio (OR) 53.8 95% CI 3.37–859), the multivariate analysis showed that age at least 65 years (OR 10.9 95% CI 2.28–51.8) and an NT-proBNP value in accordance with heart failure (OR 8.62 95% CI 1.61–46.1) were significantly related to hs-cTnT >15 ng/L (Table 3). Atrial fibrillation and eGFR showed a significant association with the level of hs-cTnT in the clinical characteristics (Table 1), as well as in the logistic univariate models (not shown). The significance disappeared after adjusting these variables for age and sex. They were therefore excluded from the final model, although the *P* values (0.073 and 0.079, respectively) indicated a tendency to association with the level of hs-cTnT. The time between onset of symptoms and examination by the GP and blood sampling was at least 10 h. Figure 1 shows the individual values of hs-cTnT in relation to age. It is also clear that samples with hs-cTnT values between 30 and 50 ng/L failed to give positive values on the POCT-cTnT instrument (Cobas h232) in contradiction to the manufacturer’s specifications (a detection limit of 30 ng/L).

**Table 1:** Clinical characteristics of 115 patients with chest pain in relation to the level of hs-cTnT

	Chest pain patients *n* = 115	hs-cTnT-T < 15 ng/L *n* = 84	hs-cTnT-T ≥ 15 ng/L *n* = 31	*P* value
Demographics				
Age, years	65 ± 14	61 ± 13	76 ± 11	<0.001
Sex, male	66 (57)	41 (49)	25 (81)	0.003
Risk factors				
Current smokers	15 (13)	12 (14)	3 (10)	0.756
Diabetes	17 (15)	9 (11)	8 (26)	0.072
Hypertension	42 (37)	25 (30)	17 (55)	0.017
Hypercholesterolaemia	34 (30)	22 (26)	12 (39)	0.250
Cardiovascular disease				
Angina pectoris	20 (17)	11 (13)	9 (29)	0.056
Previous AMI	19 (17)	12 (14)	7 (23)	0.395
Coronary revascularization	14 (12)	10 (12)	4 (13)	1.000
Stroke	4 (3.5)	3 (3.6)	1 (3.2)	1.000
Heart failure	11 (10)	4 (4.8)	7 (23)	0.008
Aortic valve disease	4 (3.5)	1 (1.2)	3 (9.7)	0.059
Renal failure clinically diagnosed	1 (0.9)	0 (0)	1 (3.2)	0.270
ECG findings				
Atrial fibrillation	12 (10)	3 (3.6)	9 (29)	<0.001
Laboratory findings				
POCT-cTnT >30 ng/L	4	0	4	NA^a^
eGFR, mL/min	68 ± 16	71 ± 15	59 ± 15	<0.001
NT-proBNP^b^	13 (11)	2 (2.4)	11 (37)	<0.001
Other				
Potential causes of increased troponin T in the absence of overt ischaemic heart disease, *n* (%)^c^	1 (0.9)	1 (1.2)	0 (0)	1.000

Values are presented as mean ± SD or number (%).

^a^Not applicable.

^b^>450 ng/L (<50 years), >900 ng/L (50–75 years), and >1,800 ng/L (>75 years).

^c^Hypertrophic cardiomyopathy or amyloidosis

**Table 2:** Diagnostic accuracy of point-of-care test for cardiac troponin T (POCT-cTnT) and high sensitivity cardiac troponin T (hs-cTnT) to find acute myocardial infarction (AMI) and the combination of AMI or unstable angina (UA)

	Sensitivity		Specificity		PPV		NPV	
	*n*	%	*n*	%	*n*	%	*n*	%
AMI								
POCT-cTnT	2/3	67	110/112	98	2/4	50	110/111	99
hs-cTnT	3/3	100	84/112	75	3/31	10	84/84	100
AMI/UA								
POCT-cTnT	2/6	33	107/109	98	2/4	50	107/111	96
hs-cTnT	5/6	83	83/109	76	5/31	16	83/84	99

Decision limit: POCT-cTnT >30 ng/L, hs-cTnT >15 ng/L.

**Table 3:** Odds ratios (OR) for 115 patients with chest pain to have hs-cTnT ≥15 ng/L using univariate and multivariate logistic regression

	Beta	Standard error	Wald	Df	*P*	OR	95% confidence interval
Age (65 years or older)	2.39	0.80	8.95	1	0.003	10.9	2.28–51.8
Sex (male)	1.11	0.59	3.54	1	0.060	3.02	0.96–9.56
NT-proBNP^a^	2.15	0.86	6.33	1	0.012	8.62	1.61–46.1
AMI or UA	3.99	1.41	7.94	1	0.005	53.8	3.37–859
Constant	−3.89	0.85	21.0	1	0.000	0.02	

^a^>450 ng/L (<50 years), >900 ng/L (50–75 years), and >1,800 ng/L (>75 years).

### Follow-up of patients with hs-cTnT levels >15 ng/L but without signs of AMI or UA

Twenty-four patients with hs-cTnT ≥15 ng/L without any signs of AMI or UA were enrolled in the follow-up after 2–3 years. Three of these patients had died, all men, at the ages of 86, 89 and 93 years (two died of heart failure and one in cardiac arrest), leaving 16 men and 5 women in the remaining group to be investigated. These 21 patients had no anamnestic or ECG signs of AMI during the follow-up. Eighteen of these patients had a diagnosis of hypertension. However, according to measurements, their blood pressure was well regulated with a mean value of 120 mm Hg systolic and 70 mm Hg diastolic. All patients had cholesterol values below the reference limit (Table 4). In seven patients, hs-cTnT was normalized but in 14 cases hs-cTnT was still >15 ng/L (Table 4). The group with persistent increased hs-cTnT was on average 8 years older, had lower eGFR and higher NT-proBNP compared with the group with normalized hs-cTnT (Table 4). Atrial fibrillation was more common in the group with hs-cTnT >15 ng/L although not statistically significant.

**Table 4:** Follow-up of 21 patients assessed for chest pain with an initial hs-cTnT level of ≥15 ng/L but no signs of AMI or UA

	hs-cTnT ≥15 ng/L without AMI or UA *n* = 21	hs-cTnT after 2–3 years		*P* value
		<15 ng/L *n* = 7	≥15 ng/L *n* = 14	
Age (years)	81 ± 9	75 ± 9	83 ± 7	0.049
Male	16 (76)	5 (71)	11 (79)	1.000
Risk factors				
Current smokers	0	0	0	
Diabetes	6 (29)	2 (29)	4 (29)	1.000
Hypertension	18 (86)	5 (71)	13 (93)	0.247
Hypercholesterolaemia	7 (33)	3 (43)	4 (29)	0.638
Cardiovascular disease				
Angina pectoris	1 (5)	1 (14)	0	0.333
Previous AMI	3 (14)	2 (29)	1 (7)	0.247
Heart failure	3 (14)	1(14)	2 (14)	1.000
Aortic valve disease	3 (14)	1 (14)	2 (14)	1.000
ECG findings				
Atrial fibrillation	8 (38)	2 (29)	6 (43)	0.656
Laboratory findings				
eGFR (mL/min)	62 ± 17	74 ± 11	56 ± 16	0.017
NT-proBNP (ng/L)^a^	440 ± 775	150 ± 338	1,245 ± 883	0.017
Cholesterol (mmol/L)	4.5 ± 1.3	4.4 ± 1.7	4.6 ± 1.2	0.754
Symptoms and findings				
Angina pectoris or dyspnoea	3 (14)	2 (29)	1 (7)	0.247
Length (cm)	173 ± 7	173 ± 10	173 ± 7	0.847
Body weight (kg)	85 ± 13	85 ± 10	84 ± 15	0.889
BMI (kg/m^2^)	28 ± 4	29 ± 5	28 ± 4	0.862
Waist circumference (cm)	106 ± 11	107 ± 11	106 ± 11	0.878
Systolic BP (mm Hg)^a^	120 ± 10	120 ± 2.5	130 ± 11	0.176
Diastolic BP (mm Hg)^a^	70 ± 6	70 ± 7	65 ± 6	0.658

Patients are divided into groups according to hs-cTnT level 2–3 years after the initial assessment.

Values are presented as mean ± SD or number (%) as appropriate.

^a^Median ± quartile deviation.

## Discussion

A considerable number of patients consulting PHC have chest pain and other symptoms suggesting AMI or UA. The challenge to distinguish between heart disease and symptoms in the thoracic area due to other causes are well known amongst GPs.

The sensitivity for hs-cTnT to find AMI or the combination of AMI and UA was 100 and 83%, respectively, i.e. substantially better than for POCT-cTnT. The specificity of hs-cTnT was lower compared with the POCT method, but was similar to the findings in a population with acute chest pain in the emergency department (ED) (75%). However, the PPV for AMI in our study was only 10% compared with 63% in the ED [19]. This emphasizes the difficulty with interpreting hs-cTnT results in conjunction with outcome. In order to investigate if the diagnostic usability of hs-cTnT could be improved, we performed a multivariate analysis to evaluate other possible explanations for an increased hs-cTnT level. Age 65 years or more was strongly correlated to a troponin T level ≥15 ng/L. This in accordance with the findings of Hammarsten et al. [12] who found highly age-dependent cTnT percentiles above a breaking point of around 65 years in ED patients without conditions that increase cTnT. In accordance with our results, several authors have suggested that a higher decision limit must be considered for patients older than 65–70 years [12, 20, 21]. In our study, male sex was at the limit of significance (Table 3). Population-based studies on people of younger ages have shown higher cTnT values for men compared with women [14]. This has also been found in one study of ED patients older than 65 years [12]. However, some recent studies have failed to show gender differences at ages above 75 years [20, 21]. We also found that NT-proBNP above a level in accordance with heart failure had a strong correlation to a cTnT level of ≥15 ng/L, whereas the clinical diagnosis of heart failure had not. It is well known that a diagnosis of heart failure based on clinical findings is very uncertain and patients are often managed without a confirming echocardiogram in PHC [22]. Hence, there may have been some undiagnosed cases of heart failure in the study. Renal function was not correlated to increased hs-cTnT according to the multivariate analyses, probably explained by the well-known influence of age on renal function.

A dynamic course of the cTnT level is an important diagnostic criteria for AMI [5]. However, a single hs-cTnT value could serve as decision support when safely ruling out AMI in patients with recent onset of symptoms or to diagnose AMI or UA with diffuse symptoms of longer duration. A limit of detection well below 15 ng/L and the cardiac specificity of cTnT is a prerequisite for ruling out AMI in patients with recent onset of symptoms [5]. However, this gain in sensitivity for AMI would be at the expense of a low or very low positive predictive value as shown in Table 2. There is no ideal POCT for the diagnosis of AMI within 6 h of onset of symptoms [23]. However, no patient in our study had a history of less than 10 h until the blood sample was drawn. After an AMI, the hs-cTnT value may remain above the decision limit of ≥15 ng/L for 2 weeks [5]. Therefore, we included all patients with onset or aggravation of symptoms during the last 7 days in order to detect all possible cases of AMI or UA. In a previous study, we found that the use of POCT-cTnT with a detection limit of 30 ng/L in primary care may occasionally result in missed diagnoses of UA or AMI, but as shown in Table 2, hs-cTnT may be a diagnostic support to find patients even with UA [11].

Although there are several POCT instruments equipped with methods to detect troponin, they all have methodological problems. Most methods for measuring troponin lack the high sensitivity of most hospital laboratory methods and some have problems with interfering substances [23, 24]. However, attempts to develop more sensitive POCT troponin assays have been made [25, 26]. The POCT-cTnT used in this study had a detection level of 30 ng/L according to the manufacturer’s specifications. However, when hs-cTnT was measured in samples drawn at the same time, a considerable number of patients had hs-cTnT levels above 30 ng/L, but failed to give positive results with the POCT-cTnT (Figure 1). This emphasizes the need for proper validation of POCT methods both in relation to the detection limit and clinical outcome.

### Follow-up of patients with hs-cTnT levels >15 ng/L but without signs of AMI or UA

After 2–3 years, two-thirds of the 21 patients still had a cTnT level ≥15 ng/L. This can be compared with the fact that ED patients, at least 75 years old and without conditions that increase cTnT, have been shown to have cTnT levels above the 99th percentile in 43–48% of cases [20, 21]. In one-third of these cases, cTnT levels had normalized after almost 3 years. Subclinical myocardial infarctions may have been present but there were no signs of q-waves on the ECG at follow-up. At the follow-up, patients with mild heart failure could possibly have had increased cTnT values at some time due to temporary infections or other causes that normalize without treatment [5]. There were more patients with atrial fibrillation, although not statistically significant, in the group with cTnT >15 ng/L. This is in accordance with previous studies showing that atrial fibrillation is associated with increased baseline cardiac troponin values [27, 28]. It is well known that normal aging affects renal function and the level of NT-proBNP [18, 29]. However, our study group was too small for a multivariate analysis aiming to adjust eGFR and NT-proBNP for age.

### Strengths and limitations

A major strength of the study is the prospective design and rigorous follow-up of patients, even if sent home, to evaluate missed diagnoses. A limitation is the low number of cases of AMI and UA influencing the reliability of the sensitivity and specificity calculations. However, this reflects the situation in Swedish primary care, where patients with acute chest pain symptoms are directed to emergency care without passing the PHC centre. A larger study population would probably lead to a clearer association between the level of hs-cTnT, atrial fibrillation and eGFR.

### Potential clinical implications

The 99th percentile used to set the limit for ruling out using the cTnT level is based on measurements in a relatively young healthy population 20–71 years of age (median 45 years) [14]. In accordance with the results in this study, several authors have suggested that a higher limit has to be considered for patients older than 65–70 years [12, 20, 21], which is in line with our findings.

Additional clinical information could be gained if NT-proBNP is analysed at the same time as hs-cTnT. Both these cardiac markers can possibly serve as tools for managing patients with cardiac disease in primary care when making the decision on whether to refer emergently for hospital care or to monitor non-acute cardiac patients.

## Conclusions

A reliable POCT hs-cTnT could become valuable in primary care if applied to patients younger than 65 years presenting with symptoms suggestive of AMI or UA. For patients older than 65–70 years a higher decision limit of cTnT than ≥15 ng/L should be considered. The POCT-cTnT instrument Cobas h232 did not perform analytically as well as specified by the manufacturer, especially in the lower measuring range. This study emphasizes that before introducing new methods for cardiac markers in primary care, the method must be validated by independent researchers and clinical outcomes should be evaluated in a large enough, typical, primary care patient population.

## Abbreviations

AMI: acute myocardial infarction
cTnT: cardiac troponin T
ECG: electrocardiogram
ED: emergency department
eGFR: estimated glomerular filtration rate
GP: general practitioner
hs-cTnT: high sensitivity cardiac troponin T
NTproBNP: N-terminal pro-B-type natriuretic peptide
PHC: primary health care
POCT: point-of-care test
UA: unstable angina
